# Correction: Sustainable process for the preparation of potassium sulfate by electrodialysis and its concentration and purification by a nanofiltration process

**DOI:** 10.1039/d0ra90115f

**Published:** 2020-11-11

**Authors:** Jaladhi S. Trivedi, Vaibhavee Bhadja, B. S. Makwana, Suresh K. Jewrajka, Uma Chatterjee

**Affiliations:** Reverse Osmosis (Membrane) Division, Central Salt and Marine Chemicals Research Institute G. B. Marg Bhavnagar Gujarat India skjewrajka@csmcri.org; Electromembrane Processes Division, Central Salt and Marine Chemicals Research Institute G. B. Marg Bhavnagar Gujarat India umac@csmcri.org vaibhavee.bhadja@gmail.com bsmakwana@csmcri.org umac@csmcri.res.in; Academy of Scientific and Innovative Research (AcSIR) Ghaziabad-201002 India

## Abstract

Correction for ‘Sustainable process for the preparation of potassium sulfate by electrodialysis and its concentration and purification by a nanofiltration process’ by Jaladhi S. Trivedi *et al.*, *RSC Adv.*, 2016, **6**, 71807–71817, DOI: 10.1039/C6RA14303B.

The authors regret that there was an error in the author affiliations given in the original article. The correct information is given in the affiliations section here.

The Royal Society of Chemistry apologises for these errors and any consequent inconvenience to authors and readers.

## Supplementary Material

